# Association of Extreme Heat Events With Hospital Admission or Mortality Among Patients With End-Stage Renal Disease

**DOI:** 10.1001/jamanetworkopen.2019.8904

**Published:** 2019-08-09

**Authors:** Richard V. Remigio, Chengsheng Jiang, Jochen Raimann, Peter Kotanko, Len Usvyat, Frank W. Maddux, Patrick Kinney, Amir Sapkota

**Affiliations:** 1 Maryland Institute for Applied Environmental Health, University of Maryland School of Public Health, College Park; 2 Research Division, Renal Research Institute, New York, New York; 3 Icahn School of Medicine, Mount Sinai Hospital, New York, New York; 4 School of Public Health, Boston University, Boston, Massachusetts

## Abstract

**Importance:**

Extreme heat events (EHEs) are increasing in frequency, duration, and intensity, and this trend is projected to continue as part of ongoing climate change. There is a paucity of data regarding how EHEs may affect highly vulnerable populations, such as patients with end-stage renal disease (ESRD). Such data are needed to inform ESRD patient management guidelines in a changing climate.

**Objectives:**

To investigate the association between EHEs and the risk of hospital admission or mortality among patients with ESRD and further characterize how this risk may vary among races/ethnicities or patients with preexisting comorbidities.

**Design, Setting, and Participants:**

This study used hospital admission and mortality records of patients with ESRD who underwent hemodialysis treatment at Fresenius Kidney Care clinics in Boston, Massachusetts; Philadelphia, Pennsylvania; or New York, New York, from January 1, 2001, to December 31, 2012. Data were analyzed using a time-stratified case-crossover design with conditional Poisson regression to investigate associations between EHEs and risk of hospital admission or mortality among patients with ESRD. Data were analyzed from July 1, 2017, to March 31, 2019.

**Exposures:**

Calendar day– and location-specific 95th-percentile maximum temperature thresholds were calculated using daily meteorological data from 1960 to 1989. These thresholds were used to identify EHEs in each of the 3 cities during the study.

**Main Outcomes and Measures:**

Daily all-cause hospital admission and all-cause mortality among patients with ESRD.

**Results:**

The study included 7445 patients with ESRD (mean [SD] age, 61.1 [14.1] years; 4283 [57.5%] men), among whom 2953 deaths (39.7%) and 44 941 hospital admissions (mean [SD], 6.0 [7.5] per patient) were recorded. Extreme heat events were associated with increased risk of same-day hospital admission (rate ratio [RR], 1.27; 95% CI, 1.13-1.43) and same-day mortality (RR, 1.31; 95% CI, 1.01-1.70) among patients with ESRD. There was some heterogeneity in risk, with patients in Boston showing statistically significant increased risk for hospital admission (RR, 1.15; 95% CI, 1.00-1.31) and mortality (RR, 1.45; 95% CI, 1.04-2.02) associated with cumulative exposure to EHEs, while such risk was absent among patients with ESRD in Philadelphia. While increases in risks were similar among non-Hispanic black and non-Hispanic white patients, findings among Hispanic and Asian patients were less clear. After stratifying by preexisting comorbidities, cumulative lag exposure to EHEs was associated with increased risk of mortality among patients with ESRD living with congestive heart failure (RR, 1.55; 95% CI, 1.27-1.89), chronic obstructive pulmonary disease (RR, 1.60; 95% CI, 1.24-2.06), or diabetes (RR, 1.83; 95% CI, 1.51-2.21).

**Conclusions and Relevance:**

In this study, extreme heat events were associated with increased risk of hospital admission or mortality among patients with ESRD, and the association was potentially affected by geographic region and race/ethnicity. Future studies with larger populations and broader geographic coverage are needed to better characterize this variability in risk and inform ESRD management guidelines and differential risk variables, given the projected increases in the frequency, duration, and intensity of EHEs.

## Key Points

**Question:**

Are extreme heat events (EHEs) associated with increased risk of hospital admission and mortality among patients with end-stage renal disease (ESRD), and does this risk differ by race/ethnicity or preexisting comorbidities?

**Finding:**

In this time-stratified case-crossover study of 7445 patients with ESRD, EHEs were associated with increased risk of same-day hospitalization and of same-day mortality among patients with ESRD. After stratifying by preexisting comorbidities, cumulative lag exposure to EHEs was associated with increased risk of mortality among patients with ESRD living with congestive heart failure, chronic obstructive pulmonary disease, or diabetes.

**Meaning:**

Management guidelines for ESRD need to take EHEs into consideration, given the increasing frequency of EHEs that are expected with ongoing climate change.

## Teaser

This study examines the association of extreme heat events in 3 northeastern US cities with hospital admission and mortality among patients with end-stage renal disease (ESRD).

## Introduction

The evidence that climate and human health are inextricably connected has been increasing during the last decade.^1,2^ To our knowledge, most studies have focused on exposure to extreme heat events (EHEs), as they are projected to increase in frequency, intensity, and duration with a changing climate.^3^ Prior studies on the effects of extreme heat have consistently shown an increased risk of hospital admission and mortality among the general population, particularly within urban areas.^4,5,6,7,8^ Urban communities may be disproportionately affected by extreme heat because of higher rates of poverty and more intense heat exposure due to the urban heat island effect,^7,8,9,10,11^ contributing to higher risk of hospital admission and mortality.^12,13,14,15,16,17,18,19^ However, to our knowledge, few studies have investigated how EHEs may affect highly vulnerable populations, such as individuals living with end-stage renal disease (ESRD), within these urban centers.

End-stage renal disease is the final stage of chronic kidney disease that causes a gradual decrease in renal function. Patients with ESRD require some form of renal replacement therapy, such as hemodialysis or kidney transplantation, to survive. In the United States, the most commonly administered form of renal replacement therapy is thrice-weekly hemodialysis treatment.^20^ Patients with ESRD must also adhere to dietary modifications, such as restricting the consumption of water and foods containing high levels of sodium, potassium, and phosphorus, to manage excess fluid accumulation.^21^ Data from 2017^22^ suggest that there were 500 000 patients with ESRD in the United States undergoing routine hemodialysis treatment in 2015, with an annual Medicare treatment and management cost of $34 billion.

Previous studies^23,24^ have reported seasonal as well as regional patterns of mortality among patients undergoing hemodialysis, with higher rates observed in the tropical regions of the world. Other studies^13,25^ have hypothesized renal-related diagnoses, such as kidney failure, and electrolyte imbalances are the underlying causes of hospital admission and mortality risk among elderly populations exposed to high temperatures. Such diagnoses are frequent sequelae of heat stress and dehydration during periods of extreme heat^25^ and have been implicated in acute renal failure among sugarcane workers working in harsh outdoor conditions.^15,16^ A 2018 study^26^ reported extended periods of exposure to extreme heat were associated with an increased risk of acute kidney injury–related emergency department visits and hospital admissions among older adults. However, to our knowledge, such associations between EHEs and hospital admission or mortality among patients with ESRD has not been characterized, and it is unclear if such associations differ by race/ethnicity or preexisting comorbidities.

We used data from the Fresenius Kidney Care (FKC) clinics to investigate the association between EHEs and risk of hospital admission or mortality among patients with ESRD undergoing hemodialysis treatment in 3 northeastern cities: Boston, Massachusetts; New York City, New York (NYC); and Philadelphia, Pennsylvania. Our objectives were to (1) quantify risk of hospital admission or mortality among patients with ESRD associated with EHEs; (2) investigate whether this risk varied by race/ethnicity or preexisting comorbidities, including congestive heart failure (CHF), diabetes, and chronic obstructive pulmonary disease (COPD); and (3) characterize the time course of mortality and hospital admission risk associated with EHEs using time-varying (lagged) exposures.

## Methods

### Health Data

**Figure 1.**
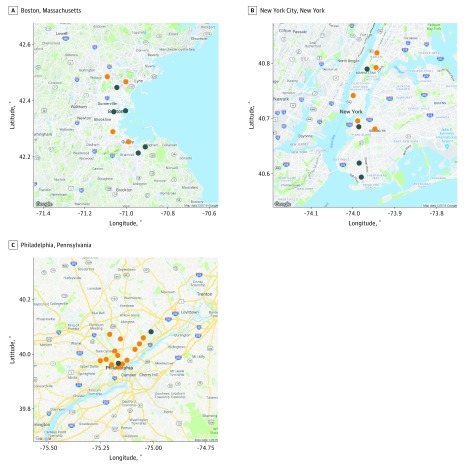
Map of Fresenius Kidney Care Hemodialysis Clinics and Weather Stations in Boston, Massachusetts; New York City, New York; and Philadelphia, Pennsylvania Blue dots indicate weather stations; orange dots, hemodialysis clinics. Map data © 2019 Google.

The period for this analysis was from January 1, 2001, to December 31, 2012, focusing on warmer months of the year (May to September). We obtained deidentified data on patients with ESRD who were treated at 23 FKC clinics (Figure 1) located in Boston, NYC, and Philadelphia from electronic health records.^27^ Eligible patients were selected based on clinic zip codes where hemodialysis treatment was received. Our study population can be considered a representative sample of patients with ESRD, who typically receive fully or nearly fully managed care for hemodialysis treatments.^28^ We used all-cause hospital admission and all-cause mortality as the primary outcomes. Counts of hospital admissions and mortality events were aggregated for each day by location. We obtained information on self-reported race/ethnicity and categorized patients as Hispanic, non-Hispanic black, non-Hispanic white, Asian, or other (eg, American Indian, Native Hawaiian, other). We also obtained information on other preexisting comorbidities, including CHF, COPD, and diabetes. Patients who had received fewer than 30 hemodialysis treatments at a given clinic were excluded to ensure location membership during the study. This study was determined exempt by the Western Institutional Review Board and the University of Maryland Institutional Review Board, which waived the need for informed consent because it used deidentified data. Our study followed the Strengthening the Reporting of Observational Studies in Epidemiology (STROBE) reporting guideline for case-control studies. Data were analyzed from July 1, 2017, to March 31, 2019.

### Extreme Heat Events

Extreme heat events were identified using previously described methods.^29,30^ In brief, we used 30 years (1960-1989) of daily meteorological data on maximum temperatures obtained from the US National Center for Environmental Information through the National Oceanic and Atmosphere Agency to calculate unique calendar day– and location-specific 95th-percentile thresholds. Following this, daily maximum temperature for targeted study locations from 2001 through 2012 were compared with their respective calendar day– and location-specific thresholds and assigned a value of 1 if they exceeded the upper 95th-percentile threshold and 0 if they did not. Days exceeding the thresholds were identified as EHEs.

### Statistical Analysis

We applied a time-stratified case-crossover study design with conditional Poisson regression to estimate location-specific associations of EHE exposures with hospital admission and mortality risk among patients with ESRD using the *gnm* package in R statistical software version 3.5.0 (R Project for Statistical Computing). In a case-crossover study design, each individual serves as his or her own control. This unique feature of the case-crossover design eliminates the need to adjust for individual level time-invariant confounders, including age, sex, race/ethnicity, and socioeconomic status.^31,32,33,34^ This study design with conditional Poisson model accounts for varying population changes during a study and allows adjustments for autocorrelation and overdispersion, which is not possible with conditional logistic regression methods.^35^ Furthermore, when adjustment for autocorrelation and overdispersion is not necessary, results obtained from conditional Poisson model are identical to those obtained with conditional logistic regression.^35^ Stratum indicators are based on the combination of year, month, and day of the week. This approach is consistent with other studies that have used case-crossover designs to measure acute health effects associated with short-term environmental exposures.^5,36,37,38,39^ We checked for autocorrelation and overdispersion and found that the assumption that variance is proportional to its mean was violated. Thus, a quasi-Poisson method was adopted into the conditional Poisson regression.

We used unconstrained same-day (lag 0), 1-day lag (lag 1), and cumulative same-day and 1-day lag (lag 0-1) exposures to EHEs during warm months (May-September) for our selected northeastern cities and for the northeast region combined. The combined data from the 3 cities were also used to conduct stratified analyses by race/ethnicity and comorbidity status to investigate if risk associated with EHEs varied by race/ethnicity (ie, Asian, non-Hispanic black, non-Hispanic white, and Hispanic) and by preexisting comorbidities (ie, CHF, COPD, and diabetes). We further tested for effect modification by location, race/ethnicity, and comorbidities using previously described methods used in similar case-crossover analyses stratified by time-invariant demographic characteristics.^40,41^ We used a Wald χ^2^ test to determine statistical significance for EHE exposure at an α level of less than 0.05.

## Results

This study included 7445 patients with ESRD from 3 cities (Table). The participants tended to be older (mean [SD] age, 61.1 [14.6] years) and men (4283 [57.5%]). Owing to data restrictions, NYC patients’ data were not available from 2001 to 2003. Philadelphia and NYC had the most non-Hispanic black patients (Philadelphia, 2558 patients [68.0%]; NYC, 1418 patients [63.3%]), whereas non-Hispanic white patients were overrepresented in Boston (1083 patients [75.2%]). Overall, the prevalence of diabetes (1710 [23.0%]) was higher compared with CHF (939 [12.6%]) and COPD (289 [3.9%]). We observed the highest 12-year mortality rate in Boston (700 deaths [48.6%]), followed by Philadelphia (1433 deaths [38.1%]), then NYC (820 deaths [36.6%]). Hospital admission can be a recurrent event among patients undergoing hemodialysis. As such, patients in this study had a mean (SD) of 6.0 (7.5) hospital admissions during the warmer months (Table). The expected number of annual EHEs based on the 95th-percentile threshold is 18.25 (365 × 0.05). During the study, the annual mean (SD) number of EHEs was higher in Boston (37.4 [23.0] days) but lower in NYC (14.2 [10.1] days) or Philadelphia (11.9 [6.5] days) (Table; eFigure in the Supplement).

**Table.** Summary Statistics for Patients With End-Stage Renal Disease From 2001 to 2012

Characteristic	No. (%)			
	Boston, Massachusetts	New York, New York	Philadelphia, Pennsylvania	Total
Clinics, No.	4	5	14	23
Patients, No.	1439	2241	3763	7445
Men	868 (60.3)	1260 (56.2)	2155 (57.3)	4283 (57.5)
Age, mean (SD), y	66.5 (14.7)	60.5 (14.2)	59.4 (14.4)	61.1 (14.6)
Race/ethnicity				
Hispanic	61 (4.2)	420 (18.7)	426 (11.3)	907 (12.2)
Non-Hispanic black	220 (16.0)	1418 (63.3)	2558 (68.0)	4196 (56.4)
Non-Hispanic white	1083 (75.2)	208 (9.28)	717 (19.1)	2108 (28.3)
Asian	50 (3.5)	84 (3.7)	50 (1.3)	184 (2.5)
Other	15 (1.0)	18 (0.8)	38 (1.0)	71 (1.0)
Comorbidities				
Congestive heart failure	369 (25.6)	206 (9.2)	364 (9.7)	939 (12.6)
Chronic obstructive pulmonary disease	90 (6.4)	63 (2.8)	134 (3.6)	289 (3.9)
Diabetes	448 (31.1)	635 (28.3)	627 (16.7)	1710 (23.0)
Health outcomes				
Mortality, No. (%)	700 (48.6)	820 (36.6)	1433 (38.1)	2953 (39.7)
Total hospital admissions, No.	8041	11 424	25 476	44 941
Hospital admissions per patient, mean (SD)	5.6 (6.8)	5.1 (6.7)	6.8 (8.2)	6.0 (7.5)
Daily maximum temperature, mean (SD) [range], °C^a^	29.3 (5.7) [7.2-38.9]	25.8 (4.7) [9.0-39.4]	27.6 (4.8) [11.1-40.0]	25.9 (5.6) [7.2-40.0]
Extreme heat events/y, mean (SD)^a^	37.4 (23.1)	14.2 (10.1)	11.9 (6.5)	21.2 (35.6)

^a^ May through September.

**Figure 2.**
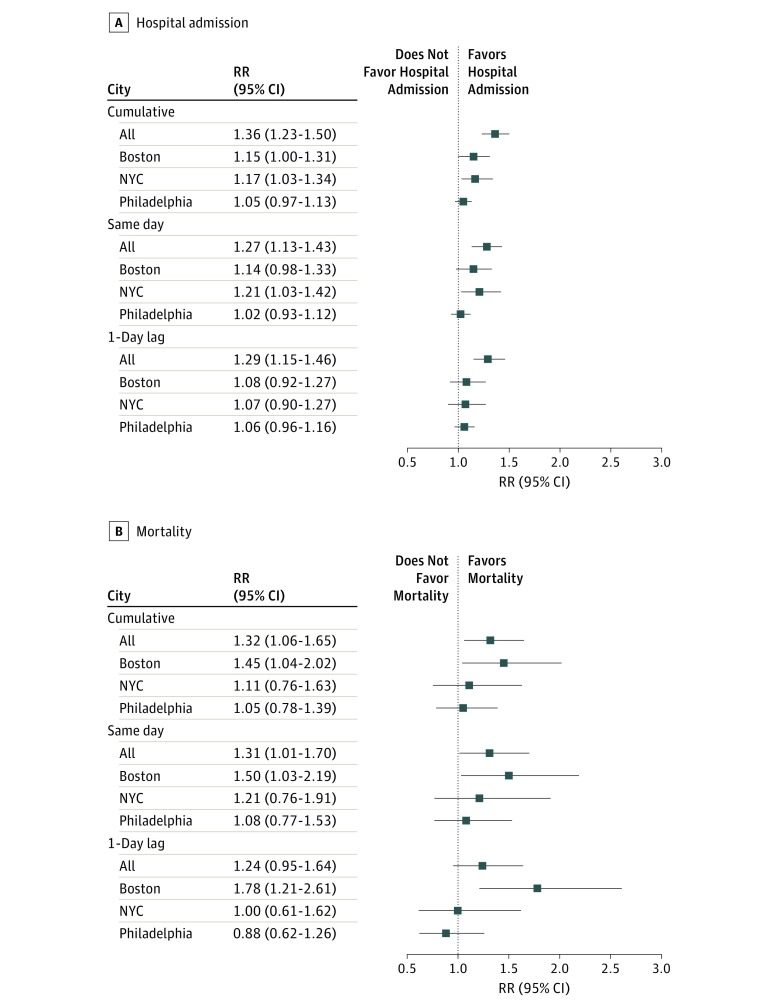
Risk of Hospital Admission and Mortality Associated with Extreme Heat Events Among Patients with End-Stage Renal Disease in Boston, Massachusetts; New York City, New York (NYC); and Philadelphia, Pennsylvania RR indicates rate ratio.

In the combined regional analysis, cumulative exposure to EHEs was associated with higher risk of hospital admission among patients with ESRD (rate ratio [RR], 1.36; 95% CI, 1.23-1.50) (Figure 2A). City-specific risks of hospital admission were statistically significant for Boston (RR, 1.15; 95% CI, 1.00-1.31) and NYC (RR, 1.17; 95% CI, 1.03-1.34) but not for Philadelphia (RR, 1.05; 95% CI, 0.97-1.13). Findings regarding same-day exposure and 1-day lagged exposure were robust for the combined analysis, but the city specific estimates were statistically significant for NYC only (RR, 1.21; 95% CI, 1.03-1.42). Likewise, cumulative exposure to EHEs was associated with increased risk of mortality among patients with ESRD in the regional analysis (RR, 1.32; 95% CI, 1.06-1.65) as well as in Boston (RR, 1.45; 95% CI, 1.04-2.02) but not in NYC (RR, 1.11; 95% CI, 0.76-1.63) or Philadelphia (RR, 1.05; 95% CI, 0.78-1.39). Findings for Boston remained statistically significant for same-day (RR, 1.50; 95% CI, 1.03-2.19) and 1-day lag (RR, 1.78; 95% CI, 1.21-2.61) exposures (Figure 2B).

**Figure 3.**
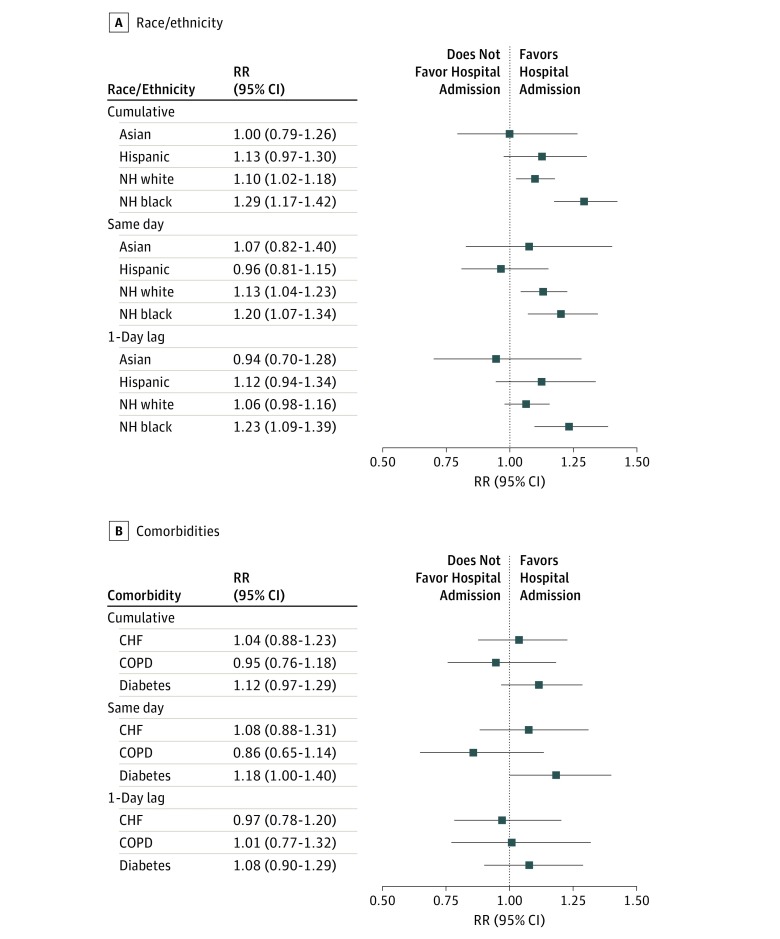
Risk of Hospital Admission Associated With Extreme Heat Events Among Patients With End-Stage Renal Disease Stratified by Race/Ethnicity and Comorbidities CHF indicates congestive heart failure; COPD, chronic obstructive pulmonary disease; NH, non-Hispanic; and RR, rate ratio.

**Figure 4.**
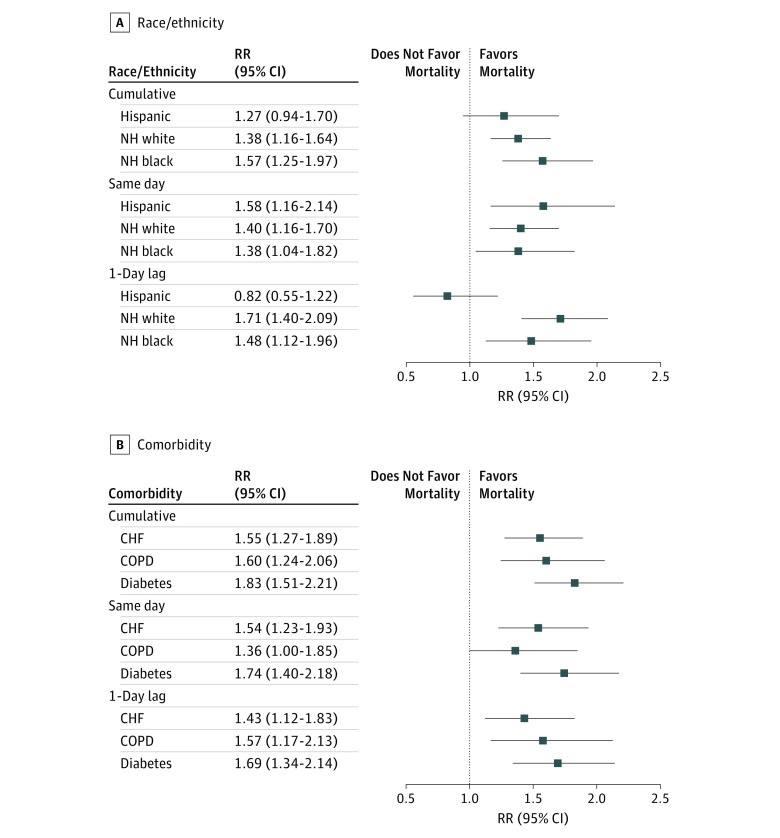
Mortality Risk Associated With Extreme Heat Events Among Patients With End-Stage Renal Disease Stratified by Race/Ethnicity and Comorbidities CHF indicates congestive heart failure; COPD, chronic obstructive pulmonary disease; NH, non-Hispanic; and RR, rate ratio.

We further stratified the analysis for hospital admission (Figure 3) and mortality (Figure 4) by race/ethnicity and for comorbidities. Cumulative exposure to EHEs was associated with increased risk of hospital admission among non-Hispanic black patients (RR, 1.29; 95% CI, 1.17-1.42) and non-Hispanic white patients (RR, 1.10; 95% CI, 1.02-1.18) but not among Hispanic patients (RR, 1.13; 95% CI, 0.97-1.30). The positive findings for non-Hispanic black and non-Hispanic white patients were consistent for same-day exposure, but the 1-day lag exposure was statistically significant only among non-Hispanic black patients (RR, 1.23; 95% CI, 1.09-1.39). Extreme heat events were not associated with risk of hospital admissions among Asian patients regardless of the lag structure (Figure 3A). Same-day EHE exposure was associated with increased risk of hospital admission among patients with diabetes as a cormorbidity (RR, 1.18; 95% CI, 1.00-1.40); however, such risk was not observed among patients with CHF or COPD as preexisting comorbidities (Figure 3B).

Cumulative exposure to EHEs was associated with increased risk of mortality among non-Hispanic black patients (RR, 1.57; 95% CI, 1.25-1.97) and non-Hispanic white patients (RR, 1.38; 95% CI, 1.16-1.64) but not among Hispanic patients (RR, 1.27; 95% CI, 0.94-1.70). The increases in risk of mortality observed among non-Hispanic black and non-Hispanic white patients remained elevated for both same-day and 1-day lag exposures. For Hispanic patients, same-day exposure to extreme heat was associated with increased risk of mortality (RR, 1.58; 95% CI, 1.16-2.14), but exposure from the previous day was associated with decreased risk of mortality, although the decrease was not statistically significant (RR, 0.82; 95% CI, 0.55-1.22) (Figure 4A). Because there were too few deaths among Asian patients with ESRD (<5 deaths) during EHEs, we removed them from the analysis owing to model instability. The increases in risk of mortality associated with EHEs were statistically significant among patients with ESRD and living with COPD (RR, 1.60; 95% CI, 1.24-2.06), CHF (RR, 1.55; 95% CI, 1.27-1.89), or diabetes (RR, 1.83; 95% CI: 1.51-2.21) (Figure 4B). Findings for same-day and previous-day EHE exposures were similar to those for the cumulative exposure. We found some evidence of effect modification by race/ethnicity and location. These findings were higher than the overall increases in mortality observed for the combined population (RR, 1.32; 95% CI, 1.06-1.65), indicating potential effect modification (Figure 2B).

## Discussion

The projected increases in the duration, frequency, and intensity of EHEs associated with climate change are a significant public health concern, as they can negatively affect vulnerable populations, such as patients with ESRD. The geographic heterogeneity in EHEs observed among Boston, NYC, and Philadelphia during the study is in agreement with previous studies^42,43^ that have noted such variability in the influence of climate change on local weather events. Our results suggest that EHEs are associated with increased risk of hospital admission and mortality among patients with ESRD. While the risk estimates for hospital admission and mortality were consistent for the regional combined analysis, city-specific risk estimates differed, highlighting the geographic variability. Increases in risk of hospital admission and mortality associated with EHEs were consistently higher among non-Hispanic black and non-Hispanic white patients, but findings among Hispanic and Asian patients were less clear. Risk of mortality associated with EHEs among patients with ESRD was consistent among patients with CHF, COPD, or diabetes as comorbidities. However, hospital admission risk differed by comorbidity status, ie, EHEs were associated with increased risk of same-day hospital admission among patients with ESRD and diabetes, but this risk was not elevated among patients with ESRD and COPD or CHF as comorbidities. These preliminary findings highlight the need for national scale assessments to quantify the underlying geographic and demographic variability in risk of hospital admission and mortality associated with EHEs to better inform ESRD management in a changing climate.

Our findings of increased hospital admission and mortality risk associated with EHEs among patients with ESRD is consistent with previous studies^13,16,44^ that have reported similar findings among general populations. While both NYC and Philadelphia had higher proportions of non-Hispanic black patients and similar rates of EHEs, neither the risk of hospital admission nor of mortality associated with EHEs was statistically significant in Philadelphia. The interpretation of this observation remains unclear, as we did not investigate facility-specific characteristics. Our finding of increased mortality associated with EHEs among patients with ESRD and CHF or COPD as comorbidities is consistent with a 2010 study^45^ among elderly people with CHF and COPD. According to the US Renal Disease Data System,^22^ cardiovascular disease, including CHF, account for almost half of all deaths among patients undergoing hemodialysis. Lowered blood pressure is a common physiological response to increasing ambient temperature among individuals irrespective of cardiovascular health status.^46,47^ However, with respect to people with ESRD, low blood pressure has been shown to increase the risk of premature death.^48,49,50^ The underlying mechanism for heat related mortality among patients with ESRD living with COPD or diabetes remains unclear and needs further investigation.^51,52^

Individual-level determinants, such as education level, socioeconomic status, and race/ethnicity, have been shown to increase vulnerability and can potentially modify the association between heat exposure and mortality.^53,54,55^ While we excluded Asian patients from the mortality analysis owing to model instability attributed to a small number of events (<5 deaths) during an EHE, others have suggested this particular group may be less vulnerable to EHE exposures.^56,57^ For example, a 2015 study^58^ reported that Asian laborers in the United States exhibited a lower rate of mortality associated with occupational heat exposure during 2000 to 2010. Interestingly, among Medicare enrollees in the United States, older Asian adults appear to experience significantly reduced rates of health care visits associated with hyperthermia.^59^ We observed some evidence of effect modification by location, race/ethnicity, and comorbidities, but results were not statistically significant because of limited sample size. This is consistent with a previous study that reported higher risk of mortality among individuals with CHF, diabetes, or COPD during the summer season.^54^

### Strengths and Limitations

This study consisted of a relatively large sample size of patients with ESRD, a previously understudied vulnerable population in the context of climate change. Hospital admission and mortality records for the study population were maintained by FKC, a globally known hemodialysis care enterprise. We also computed a robust exposure metric using location- and calendar day–specific thresholds that incorporated local climatological measures. This enabled us to use EHE frequency as an exposure metric, which may be more relevant than daily mean temperature in the context of climate change.^29^ We used a time-stratified case-crossover design with conditional Poisson analysis, as it is more flexible in estimating acute effects associated with short-term exposures. Through self-matching, this study design negated the need to control for individual-level measured and unmeasured time-invariant confounders.^32,33^

The study had limitations as well. We used a single-day exposure metric; therefore, we did not account for alternative definitions for EHEs, including heat wave, as seen in other work.^13,18^ The consideration of multiple-day heat waves could represent a more severe exposure experience for patients with ESRD, especially given the frequent occurrence of heat waves in the United States. Our exposure metric was dichotomous (ie, presence or absence); thus, it did not account for the intensity of exposure on a continuous scale. In addition, a limitation of this study is the lack of data verifying indoor conditions for patients in our study population. Prior studies have reported that outdoor temperatures correlate well with indoor temperatures, especially during warmer months, in Boston^10^ and NYC.^11,60^ This suggests that extreme heat measured outdoors can serve as a surrogate for indoor environments. Another limitation is the spatial heterogeneity in exposure that may exist among the patient’s residence, the treating clinic, and the nearest weather station. Built-environment features, such as green^4,61^ and blue^62^ spaces and impervious cover,^37,63^ can influence local surface temperatures. However, potential exposure misclassification errors that resulted from the use of central weather stations were likely nondifferential in nature, as monitoring stations did not change between the case period and the control periods.^64^ This nondifferential measurement error, if present, likely attenuated the risk estimates.^65^ In this analysis, we did not adjust for time-varying confounders, such as air quality. Another limitation relates to the lack of specific causes for hospital admission and mortality for the study population. Future studies with larger sample sizes are needed to investigate how cause-specific mortality and hospital admission associated with EHEs among patients with ESRD varies by geographic locations, race/ethnicity, socioeconomic status, and comorbidities while accounting for time-varying confounders, such as air pollution.

## Conclusions

Our results showed that EHEs were associated with increased risk of hospital admission or mortality among patients with ESRD and that such risks may vary by city, race/ethnicity, and comorbidity. With the projected increases in frequency, duration, and intensity of extreme weather events, future ESRD management guideline need to incorporate EHEs as part of the adaptation measures to minimize morbidity and mortality among patients with ESRD in a changing climate.

## Notes

Supplement. **eFigure.** Yearly Total Extreme Heat Events in May Through September by City
