# Supplementary material for: Association of Extreme Heat Events With Hospital Admission or Mortality Among Patients With End-Stage Renal Disease
Source: JAMA Netw Open. 2019 Aug 9;2(8):e198904. doi: 10.1001/jamanetworkopen.2019.8904 (PMC6692691; doi:10.1001/jamanetworkopen.2019.8904)
Supplement: Supplement. — eFigure. Yearly Total Extreme Heat Events in May Through September by City [file jamanetwopen-2-e198904-s001.pdf]

Supplementary Online Content

Remigio RV, Jiang C, Raimann J, et al. Association of extreme heat events with hospital admission or mortality among patients with end-stage renal disease. *JAMA Netw Open*. 2019;2(8):e198904. doi:10.1001/jamanetworkopen.2019.8904

eFigure. Yearly Total Extreme Heat Events in May Through September by City

This supplementary material has been provided by the authors to give readers additional information about their work.

© 2019 Remigio RV et al. *JAMA Network Open.*

eFigure. Yearly Total Extreme Heat Events in May Through September by City

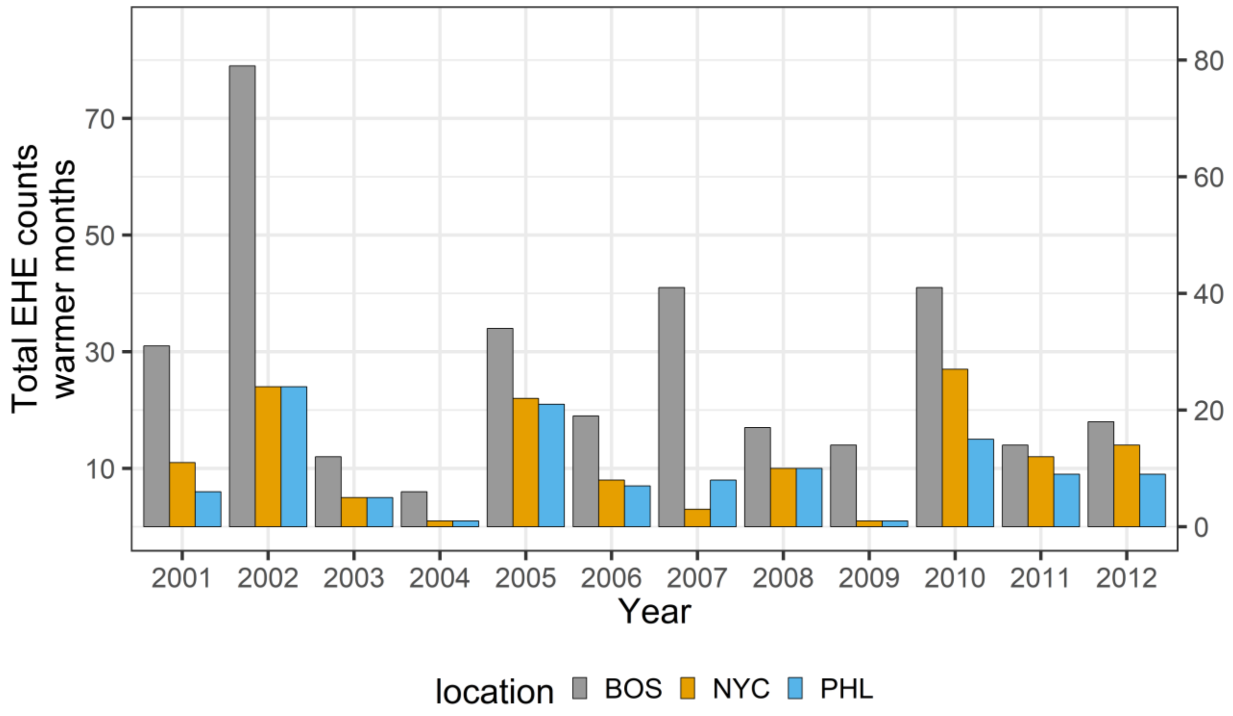

© 2019 Remigio RV et al. *JAMA Network Open*.
